# Supplementary material for: Weaning drives microbiome-mediated epigenetic regulation to shape immune memory in mice
Source: Nat Microbiol. 2026 Mar 19;11(4):1064–79. doi: 10.1038/s41564-026-02295-6 (PMC13056565; doi:10.1038/s41564-026-02295-6)
Supplement: Supplementary file 1 — Supplementary Figs. 1 and 2. [file 41564_2026_2295_MOESM1_ESM.pdf]

nature microbiology

Supplementary information https://doi.org/10.1038/s41564-026-02295-6

Weaning drives microbiome-mediated epigenetic regulation to shape immune memory in mice

In the format provided by the authors and unedited

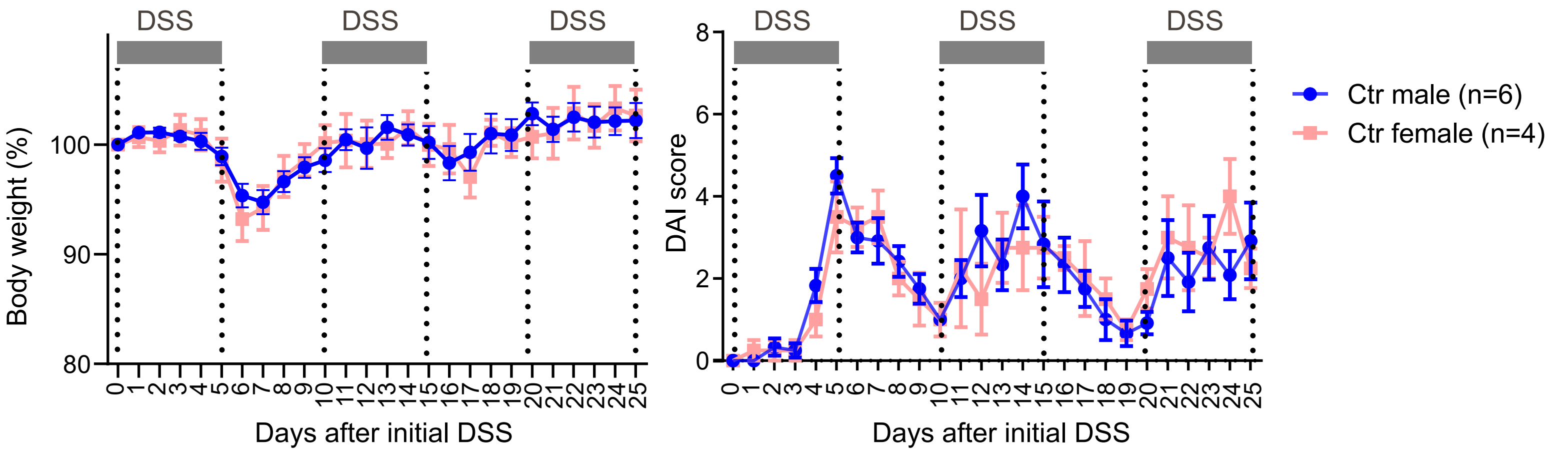

Supplementary Information Figure 1: Comparable DSS responses in male and female control mice. Body weight loss and disease activity index (DAI) during DSS treatment showed no significant differences between male and female control mice.

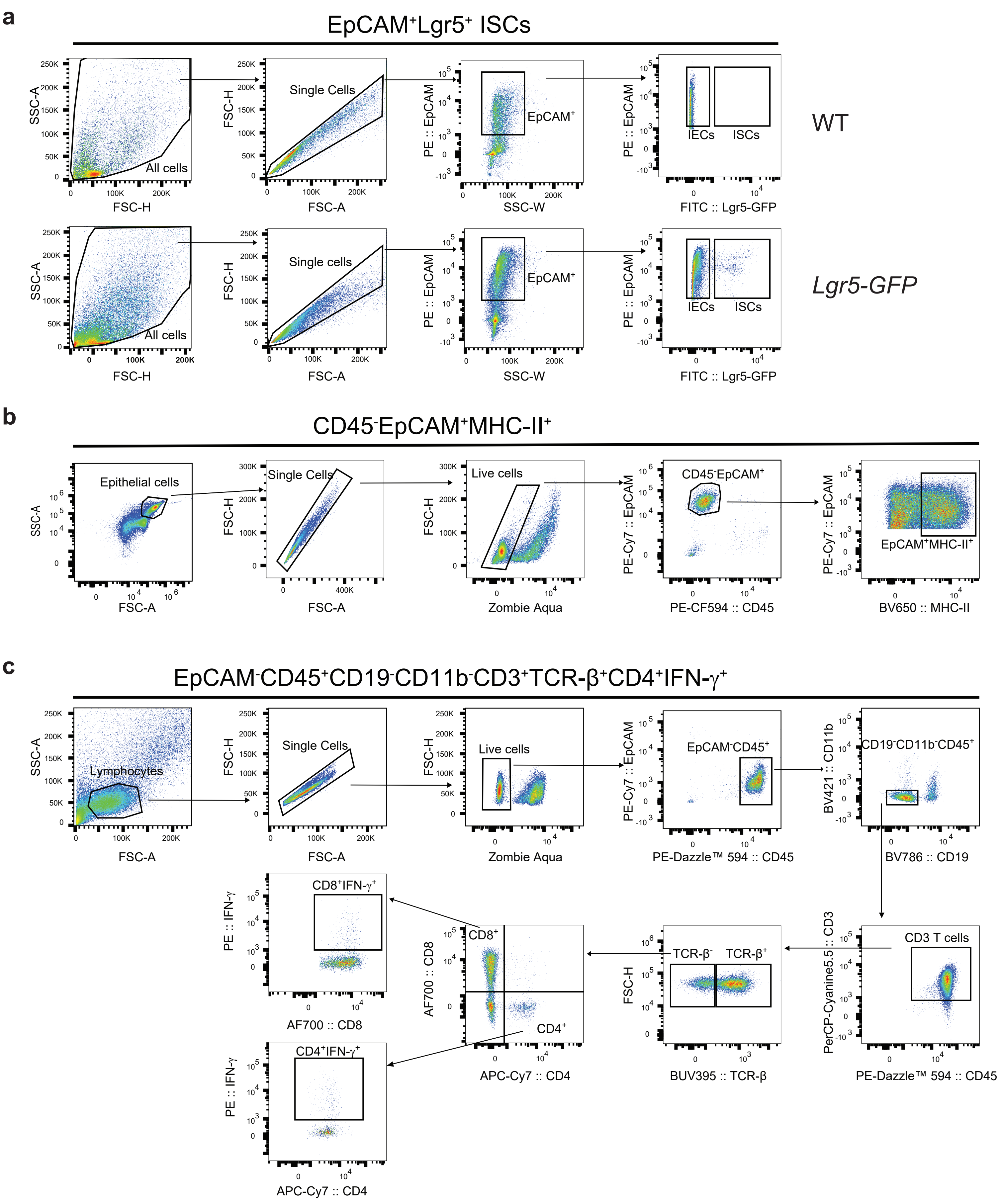

Supplementary Information Figure 2: Flow cytometry analysis and gating strategy.
(a) Flow cytometry analysis of EpCAM+Lgr5-GFP+ intestinal stem cells (ISCs).
(b) Flow cytometry analysis of CD45-EpCAM+MHC-II+ intestinal epithelial cells (IECs).
(c) Flow cytometry analysis of CD4+IFN- + cells in intraepithelial lymphocytes (IELs).
