# Supplementary material for: Weaning drives microbiome-mediated epigenetic regulation to shape immune memory in mice
Source: Nat Microbiol. 2026 Mar 19;11(4):1064–79. doi: 10.1038/s41564-026-02295-6 (PMC13056565; doi:10.1038/s41564-026-02295-6)
Supplement: Supplementary file 2 — Reporting Summary [file 41564_2026_2295_MOESM2_ESM.pdf]

nature portfolio

Corresponding author(s): Jiejun Shi, Lanlan Shen

Last updated by author(s): Feb 6, 2026

Reporting Summary

Nature Portfolio wishes to improve the reproducibility of the work that we publish. This form provides structure for consistency and transparency in reporting. For further information on Nature Portfolio policies, see our Editorial Policies and the Editorial Policy Checklist.

Statistics

For all statistical analyses, confirm that the following items are present in the figure legend, table legend, main text, or Methods section.

n/a	Confirmed	
☐	☒	The exact sample size (n) for each experimental group/condition, given as a discrete number and unit of measurement
☐	☒	A statement on whether measurements were taken from distinct samples or whether the same sample was measured repeatedly
☐	☒	The statistical test(s) used AND whether they are one- or two-sided<br>*Only common tests should be described solely by name; describe more complex techniques in the Methods section.*
☒	☐	A description of all covariates tested
☐	☒	A description of any assumptions or corrections, such as tests of normality and adjustment for multiple comparisons
☐	☒	A full description of the statistical parameters including central tendency (e.g. means) or other basic estimates (e.g. regression coefficient) AND variation (e.g. standard deviation) or associated estimates of uncertainty (e.g. confidence intervals)
☐	☒	For null hypothesis testing, the test statistic (e.g. F, t, r) with confidence intervals, effect sizes, degrees of freedom and P value noted<br>*Give P values as exact values whenever suitable.*
☒	☐	For Bayesian analysis, information on the choice of priors and Markov chain Monte Carlo settings
☒	☐	For hierarchical and complex designs, identification of the appropriate level for tests and full reporting of outcomes
☒	☐	Estimates of effect sizes (e.g. Cohen's d, Pearson's r), indicating how they were calculated

Our web collection on statistics for biologists contains articles on many of the points above.

Software and code

Policy information about availability of computer code

Data collection	Data were saved as Microsoft Excel (.xls) and Word (.docx), Portable Document Format (.pdf), Joint Photographic Experts Group (.jpg), and Tag Image File Format (.tif). Some raw data were collected using Flow Cytometry Standard (.fcs). Genomic sequencing data were stored in raw fastq files.
Data analysis	We utilized the following publically available softwares:<br>BSMAP<br>BASALkit (http://www.github.com/JiejunShi/BASAL)<br>Metilene v0.2-862<br>RSEM<br>DEseq2<br>PyroMark Q48 Auto<br>StepOne Software V2.3<br>CellSensStandard<br>FlowJo<br>GraphPad Prism 9<br>Bowtie2 (v2.5.4)<br>Kraken2 (v2.1.3)<br>Bracken (v2.9)<br>DIAMOND (v2.1.9)

For manuscripts utilizing custom algorithms or software that are central to the research but not yet described in published literature, software must be made available to editors and reviewers. We strongly encourage code deposition in a community repository (e.g. GitHub). See the Nature Portfolio guidelines for submitting code & software for further information.

Data

Policy information about availability of data

All manuscripts must include a data availability statement. This statement should provide the following information, where applicable:

- Accession codes, unique identifiers, or web links for publicly available datasets
- A description of any restrictions on data availability
- For clinical datasets or third party data, please ensure that the statement adheres to our policy

Sequencing data are available at the GEO repository under the accession numbers "GSE275219", "GSE275418" and "GSE310995". Source data are provided in the paper.

Research involving human participants, their data, or biological material

Policy information about studies with human participants or human data. See also policy information about sex, gender (identity/presentation), and sexual orientation and race, ethnicity and racism.

Reporting on sex and gender	Our study does not involve human subjects.
Reporting on race, ethnicity, or other socially relevant groupings	Our study does not involve human subjects.
Population characteristics	Our study does not involve human subjects.
Recruitment	Our study does not involve human subjects.
Ethics oversight	Our study does not involve human subjects.

Note that full information on the approval of the study protocol must also be provided in the manuscript.

Field-specific reporting

Please select the one below that is the best fit for your research. If you are not sure, read the appropriate sections before making your selection.

☒ Life sciences ☐ Behavioural & social sciences ☐ Ecological, evolutionary & environmental sciences

For a reference copy of the document with all sections, see nature.com/documents/nr-reporting-summary-flat.pdf

Life sciences study design

All studies must disclose on these points even when the disclosure is negative.

Sample size	Sample size and statistical testes for each experiment are stated in the manuscript and figure legends. The number of animals chosen is based on our previous epigenome studies (PMID: 26420038).
Data exclusions	No data was excluded.
Replication	At least two independent experiments with three technical repeats were performed.
Randomization	Mice were randomly assigned to treatment groups. For all experiments, sample processing and data analysis were performed blinded to group assignment.
Blinding	Histology images were scored by a blinded pathologist.

Reporting for specific materials, systems and methods

We require information from authors about some types of materials, experimental systems and methods used in many studies. Here, indicate whether each material, system or method listed is relevant to your study. If you are not sure if a list item applies to your research, read the appropriate section before selecting a response.

Materials & experimental systems

n/a	Involved in the study
☐	☒ Antibodies
☐	☒ Eukaryotic cell lines
☒	☐ Palaeontology and archaeology
☐	☒ Animals and other organisms
☒	☐ Clinical data
☒	☐ Dual use research of concern
☒	☐ Plants

Methods

n/a	Involved in the study
☒	☐ ChIP-seq
☐	☒ Flow cytometry
☒	☐ MRI-based neuroimaging

Antibodies

Antibodies used

PE/Cyanine7 anti-mouse CD326 (EpCAM) (BioLegend, Clone G8.8, #118215, 1:200)
Anti-mouse CD16/CD32 (BioLegend, Clone S17011E, #156603, 1:100)
PE/Dazzle™ 594 anti-mouse CD45 (BioLegend, Clone 30-F11, #103145, 1:100)
BUV395 hamster anti-mouse TCRβ (BD, Clone H57-597, #569248, 1:100)
PerCP/Cyanine5.5 anti-mouse CD3 (BioLegend, Clone 17A2, #100217, 1:50)
APC/Cy7 anti-mouse CD4 (BioLegend, Clone RM4-5, #100525, 1:50)
Alexa Fluor® 700 anti-mouse CD8a (BioLegend, Clone 53-6.7, #100729, 1:200)
Brilliant Violet 785™ anti-mouse CD19 (BioLegend, Clone 6D5, #115543, 1:100)
Brilliant Violet 421™ anti-mouse CD11b (BioLegend, Clone M1/70, #101235, 1:50)
Brilliant Violet 650™ anti-mouse I-A/I-E (MHC II) (BioLegend, Clone M5/114.15.2, #107641, 1:100)
PE anti-mouse IFN-γ (BioLegend, Clone XMG1.2, #505807, 1:50)
PE anti-mouse CD326 (EpCAM) antibody (Biolegend, Clone G8.8, #118206, 1:200)
APC anti-mouse CD24 (BioLegend, Clone 30-F1, #138505, 1:100)
Ultra-LEAF™ purified anti-mouse IFN-γ antibody (BioLegend, Clone XMG1.2, #505847)
Ultra-LEAF™ purified Rat IgG1, κ Isotype Ctrl antibody (BioLegend, Clone RTK2071, #400457)
TET1 polyclonal antibody [N1], N-term (GeneTex, #GTX125888, 1:50)
TET2 Rabbit Monoclonal Antibody (Cell Signaling Technology, Clone D9K3E, #92529, 1:25)
Anti-TET3 polyclonal antibody (Sigma-Aldrich, #ABE290, 1:50)
Stat1 polyclonal antibody (Cell Signaling Technology, #9172, 1:25)
Stat3 rabbit monoclonal antibody (Cell Signaling Technology, Clone D3Z2G, #12640, 1:25)
Normal Rabbit IgG (Cell Signaling Technology, #2729S, 1:500)
Ki-67 recombinant rabbit monoclonal antibody (ThermoFisher Scientific, clone SP6, #MA5-14520, 1:100)
Anti-CD3 antibody (Abcam, Clone SP7, #ab16669, 1:100)
Horse anti-rabbit IgG antibody (H+L), Biotinylated (Vector Laboratories, BA-1100-1.5, 1:200)

Validation

https://www.biolegend.com/nl-be/quality/quality-assurance-certificates
https://www.genetex.com/Info/guarantee
https://www.cellsignal.com/about-us/cst-antibody-validation-principles
https://www.sigmaaldrich.com/US/en/life-science/quality-and-regulatory-management
https://www.thermofisher.com/us/en/home/life-science/antibodies/invitrogen-antibody-validation
https://www.abcam.com/en-us/stories/articles/biophysical-quality
https://vectorlabs.com/immunohistochemistry/

Eukaryotic cell lines

Policy information about cell lines and Sex and Gender in Research

Cell line source(s)	HEK-293T cells were purchased from ATCC.
Authentication	Authentication was assessed by short tandem repeats (STR) profiling.
Mycoplasma contamination	Cells were tested negative for mycoplasma contamination.
Commonly misidentified lines (See ICLAC register)	No commonly misidentified cell lines were used in this study.

Animals and other research organisms

Policy information about studies involving animals; ARRIVE guidelines recommended for reporting animal research, and Sex and Gender in Research

Laboratory animals	Lgr5-EGFP-IRES-CreERT2 mice (Jackson Laboratory, Strain # 008875) were backcrossed with C57BL/6J mice for over 20 generations to ensure a genetically identical background.
Wild animals	This study did not involve wild animals.

Reporting on sex	Both male and female mice were used across experiments.
Field-collected samples	Specific pathogen-free (SPF) and germ-free (GF) housing conditions were employed. Routine serological tests were conducted to exclude the presence of specific pathogens. The GF mice were monitored monthly using 16S sequencing.
Ethics oversight	All experiments were conducted with approval from the Institutional Animal Care and Use Committee (IACUC) at Baylor College of Medicine (protocol number AN-6775).

Note that full information on the approval of the study protocol must also be provided in the manuscript.

Plants

Seed stocks	Not applicable
Novel plant genotypes	Not applicable
Authentication	Not applicable

Flow Cytometry

Plots

Confirm that:

☒ The axis labels state the marker and fluorochrome used (e.g. CD4-FITC).

☒ The axis scales are clearly visible. Include numbers along axes only for bottom left plot of group (a 'group' is an analysis of identical markers).

☒ All plots are contour plots with outliers or pseudocolor plots.

☒ A numerical value for number of cells or percentage (with statistics) is provided.

Methodology

Sample preparation	After dissociation with EDTA and TrypLE, the colonic epithelial cells were in single cell suspension and were incubated with anti-mouse CD326 (EpCAM) antibody. The cells were then washed, filtered, and sorted based on their expression of EpCAM and GFP: stem cells were identified as EpCAM+/GFP+ and differentiated cells were identified as EpCAM+/GFP-.<br><br>Intraepithelial lymphocytes (IELs) were enriched using 40%/80% discontinuous Percoll gradient centrifugation. For surface marker staining, IELs were first treated with Zombie Aqua™ Fixable Viability Kit for live/dead staining. Following this, cells were blocked with anti-mouse CD16/CD32 to reduce non-specific binding. Surface markers were then stained using fluorochrome-conjugated antibodies. For intracellular staining, cells were fixed using the Foxp3/Transcription Factor Staining Buffer Set according to the manufacturer's instructions. After fixation, the cells were stained with cytokine-specific antibodies.
Instrument	BD Aria Fusion cell sorter and BD Symphony A5 flow cytometer
Software	FlowJo software
Cell population abundance	At least 100000 cells were acquired for each condition.
Gating strategy	Gating strategy to identify individual cell populations are available in Supplementary Fig. 2.

☒ Tick this box to confirm that a figure exemplifying the gating strategy is provided in the Supplementary Information.
