# Supplementary figures and images for: Weaning drives microbiome-mediated epigenetic regulation to shape immune memory in mice
Source: Nat Microbiol. 2026 Mar 19;11(4):1064–79. doi: 10.1038/s41564-026-02295-6 (PMC13056565; doi:10.1038/s41564-026-02295-6)

Figure 1a

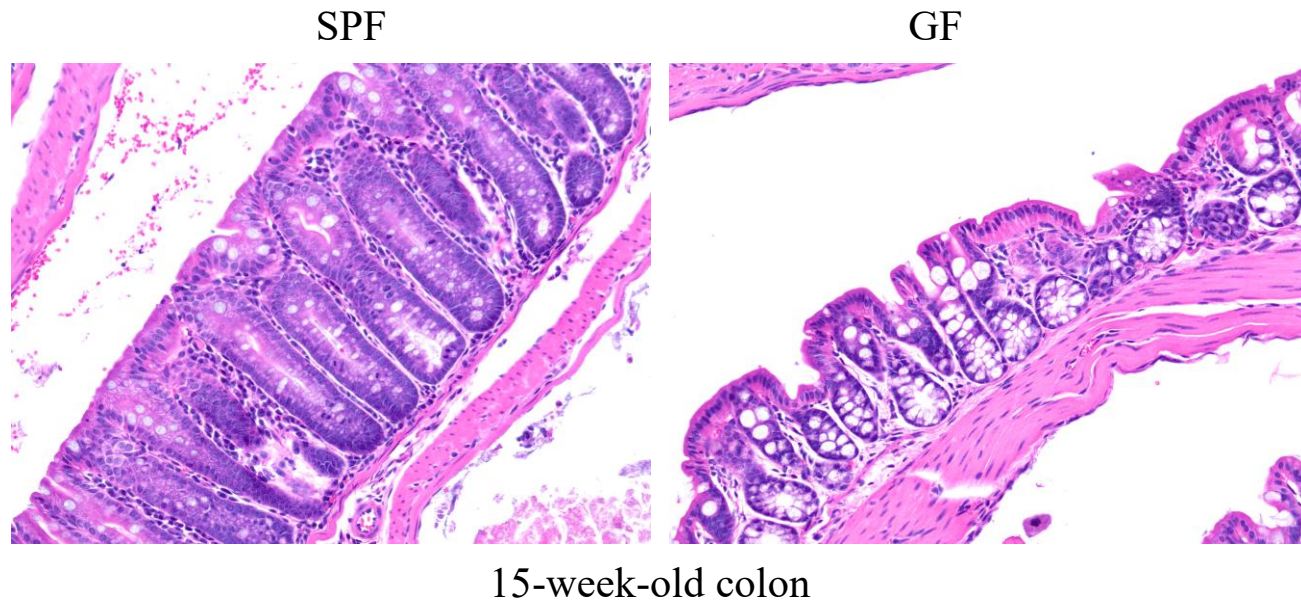

Supplement: Supplementary file 21 — Full-size histology image. [file 41564_2026_2295_MOESM21_ESM.pdf]

Extended Data Figure 1a

Ki67

Alcian Blue (AB)

SPF

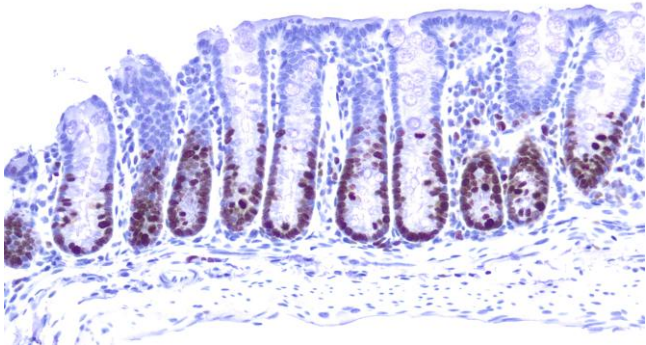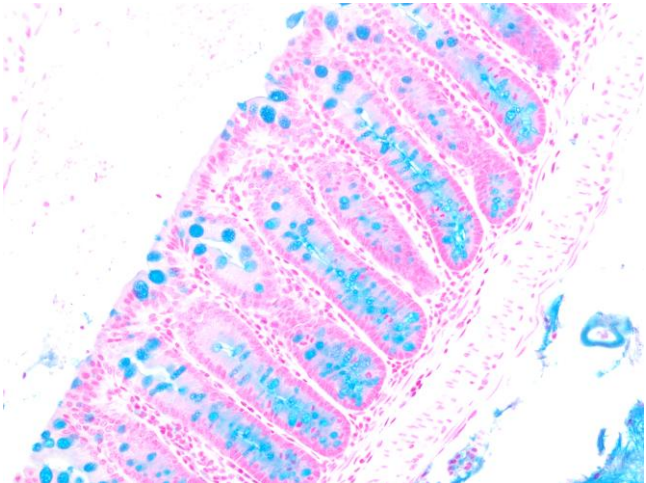

GF

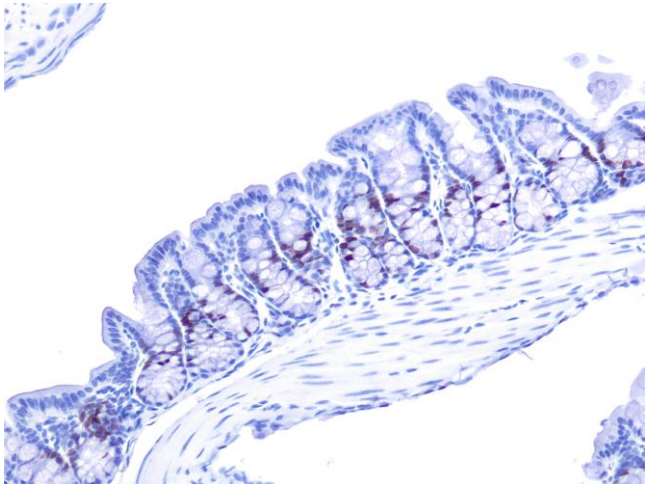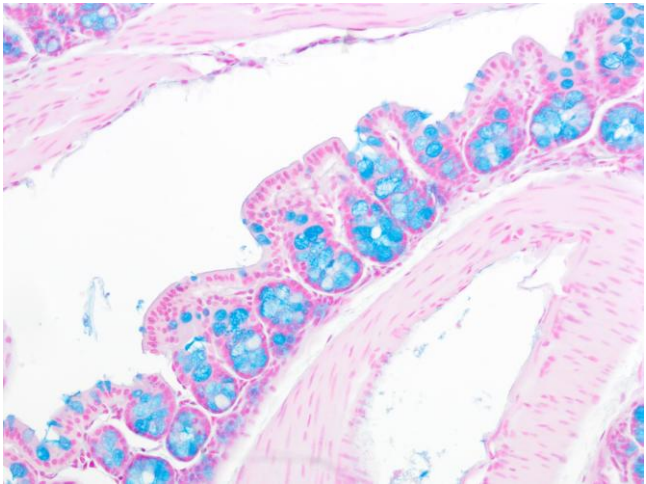

Supplement: Supplementary file 22 — Full-size histology image. [file 41564_2026_2295_MOESM22_ESM.pdf]
